# Samply Stream API: The AI-enhanced method for real-time event data streaming

**DOI:** 10.3758/s13428-025-02634-1

**Published:** 2025-03-17

**Authors:** Yury Shevchenko, Ulf-Dietrich Reips

**Affiliations:** Research Methods, Assessment, and iScience; Department of Psychology, University of Konstanz, Universitätsstraße 10, Box 31, 78464 Konstanz, Germany

**Keywords:** Experience sampling method, Real-time data streaming, Samply Stream API, Mobile surveys, AI-enhanced method

## Abstract

This manuscript introduces a novel method for conducting behavioral and social research by streaming real-time information to participants and manipulating content for experimental purposes via AI. We present an extension of the Samply software, which facilitates the integration of event-related data with mobile surveys and experiments. To assess the feasibility of this method, we conducted an experiment where news headlines were modified by a Chat-GPT algorithm and streamed to participants via the Samply Stream API and mobile push notifications. Feedback from participants indicated that most did not experience technical problems. There was no significant difference in readability across original, paraphrased, and misinformation-injected news conditions, with only 1.2% of all news items reported as unreadable. Participants reported significantly less familiarity with misinformation-injected news (84% unfamiliarity) compared to original and paraphrased news (73% unfamiliarity), suggesting successful manipulation of information without compromising readability. Dropout and non-response rates were comparable to those in other experience sampling studies. The streaming method offers significant potential for various applications, including public opinion research, healthcare, marketing, and environmental monitoring. By enabling the real-time collection of contextually relevant data, this method has the potential to enhance the external validity of behavioral research and provides a powerful tool for studying human behavior in naturalistic settings.

## Introduction

In this manuscript, we present a new research method to stream and on-the-fly experimentally manipulate event-related information to participants in real time. This streaming method can be used by behavioral and social scientists who want to collect survey data related to a specific event, such as a monarch’s funeral, a news report or a post on a social network. For example, researchers studying the perception of fake news often ask participants to evaluate a series of headlines (Bago et al., 2020). These headlines are usually from past news stories and may be outdated or familiar to participants. In contrast, evaluating real-time information using the streaming method can provide more insight into how people perceive news and make decisions in everyday contexts. This should improve external validity and allow testing existing theories outside the laboratory. We also present AI-based algorithms that can change the event stream on the fly and thereby allow for the implementation of conditions in experimental research. The method can also be used in public opinion research (Murphy et al., 2014), where real-time information can be blended with predefined survey questions (e.g., Maier et al., 2016). Finally, the streaming method can be applied to validate already existing computer algorithms that aim to detect specific events, such as rumors (Zhang et al., 2015) or fake news (Shu et al., 2017). This approach enables a hybrid verification system where content flagged by automated detection systems can be subsequently directed to professional fact-checkers for thorough human assessment and verification. The present manuscript focuses on the new method and presents a tool that we implemented to make the method widely available. Behavioral and social scientists are free to adapt this tool to their needs.

The origin of this novel method is in the experience sampling methodology, or ESM, also called “ambulatory assessment”, “ecological momentary assessment” or “diary studies” (Bolger et al., 2003; Mehl & Conner, 2011; Shiffman et al., 2008; Trull & Ebner-Priemer, 2013). ESM studies aim to examine people’s behavior and thoughts in their natural environment (Hektner et al., 2007; Larson & Csikszentmihalyi, 2014). This approach enhances external validity by capturing participants’ responses within specific situations and is particularly suitable for studying time-varying or context-dependent phenomena (e.g., mood, social interactions, developmental processes). While ESM implementation has been simplified by technological advances and widespread smartphone availability, it still requires careful preparation and management by researchers. The method’s complexity extends to data analysis, which must account for both between-subject and within-subject variability—a more demanding approach than traditional cross-sectional studies that primarily focus on between-subject comparisons. Despite these methodological challenges, the use of experience sampling in research continues to grow (Fritz et al., 2024).

With regard to the implementation of the method, participants nowadays often use their smartphones, where they can receive signals (e.g., push notifications) and provide responses. ESM signals can be sent at randomly chosen times or time intervals or depending on an event of interest (Bolger & Laurenceau, 2013; Christensen et al., 2003). With the event-contingent design, participants are asked to respond each time a specific event occurs to them. Among all possible events, we can distinguish between participant-specific and public events on the one hand and non-detectable and detectable events on the other (see Table 1).

**Table 1:** Examples of events

	*Public*	*Participant-specific*
*Detectable*	News, weather, stock market, posts in social networks	Information from smartphone sensors (e.g., location, acceleration)
*Not directly detectable*	Mood of the crowd	Urge to smoke

This article focuses on public events that an external observer can detect. Examples of detectable public events can be: a press release, a published blog, a weather change, or a stock market event. Other types of events, which we will briefly describe here, are outside of the scope of the current study, although they also contain potentially fruitful research ideas. Participant-specific detectable events can be captured by smartphone sensors. For example, a smartphone can track when participants enter or exit a predefined geographical area (e.g., geofencing in Shevchenko & Reips, 2023). Studying non-detectable participant-specific events, such as the urge to smoke, can be done by instructing participants to report this event when it happens. However, self-initiating the report is associated with a number of problems, such as social desirability or simply forgetting to report an event. With regard to not directly detectable public events, there is limited literature. One approach has been to rely on subjective judgments to measure some public event (e.g., crowd mood in Bucher & Voss, 2019).

This paper presents software that allows notifying a study participant via a smartphone application at the moment when a detectable public event has occurred. The notification combined with an online survey or experiment can be used to gather data with respect to the event. This technology is implemented through an Application Programming Interface of Samply (Samply Stream API), a website and mobile application developed in our iScience research group (Shevchenko et al., 2021). Additionally, the event-related information can be manipulated in real time using AI tools, such as a Chat-GPT algorithm (OpenAI, 2023). The AI capability opens up interesting possibilities for dynamically modifying and personalizing event content.

In the following, we briefly describe Samply and the ESM designs supported by the application. We then address the event-contingent design and describe the Samply Stream API and AI algorithms that we used to manipulate the event information. Streaming event-related information in real time via the Samply Stream API provides a way to integrate detectable public events with surveys, experience sampling, and experimental research. To the best of our knowledge, this integration has not been implemented before. Therefore, we outline the possibilities and potential research questions that can be answered with the streaming method. We then describe a feasibility study that we have conducted to demonstrate how combining the streaming method with AI manipulation can be used to study the perception of news.

## Samply application

Samply is a website and a mobile application, both available free of charge to participants and researchers. The mobile application “Samply Research” for participants can be downloaded from the Apple and Google app stores. Researchers can set up a study and schedule and manage notifications using the website’s interface (available at https://samply.uni-konstanz.de/). Researchers are flexible in using their preferred tools (e.g., lab.js, Qualtrics, WEXTOR.eu, etc.) to create online surveys or experiments, whereas Samply enhances this process by sending mobile notifications via its app, directing participants straight to an online study in their mobile web browsers. Among other features, Samply records the history of interactions with notifications for further analysis.

Researchers conducting an ESM study with Samply can combine three types of experimental design: interval-, signal-, and event-contingent (Shevchenko & Reips, 2022). The interval-continent design implies that participants receive notifications at regular intervals, such as every 3 h or every day at 9:00 p.m. The signal-contingent design allows a notification to be sent randomly within a specified period (e.g., between 9:00 and 11:00 a.m.). Both interval- and signal-contingent designs are supported in Samply, with additional features such as using a local time zone or randomizing the time for each participant. Regarding event-contingent design, researchers can use the geofencing method (Shevchenko & Reips, 2023), which allows sending notifications in response to the participant entering or leaving an area (e.g., entering a store or office).

However, the vast flexibility for event-contingent designs is realized via the Samply Stream Application Programming Interface (API), a novel part of the software presented in this manuscript. The Samply Stream API is an example of a REST API that uses HTTP requests to access, use, and edit data. A REST API (Representational State Transfer Application Programming Interface) is a standardized way for applications to communicate over the Internet using HTTP methods (GET, POST, PUT, DELETE). It allows systems to exchange data through specific URLs, typically in JSON format, making it simple for client applications to interact with server-side services (Masse, 2011). The Samply Stream API allows researchers to trigger a notification for participants by sending an HTTP POST request to the Samply server. Via this mechanism, external events can initiate notifications for study participants. The main requirement is that participants download and install the Samply Research app on their smartphones, allow notifications from the app, and join a study via the app interface. For notifications to be delivered, the participant needs to have mobile Internet or be connected to Wi-Fi. Internet connectivity is also required to complete a survey or access online study materials, which researchers can link to the notification. On the researcher’s side, the setup involves creating a study on the Samply website and connecting the Samply Stream API to external events. The HTTP POST request (about ten lines of code, see Appendix A) can be embedded in other software and implemented in most programming languages or environments (e.g., JavaScript, Python, Java, cURL). Thus, the Samply Stream API is agnostic to the software or type of external interface being used to send a request. Next, we describe different ways to send a request to the Samply Stream API: from within a survey or experiment, or via a publicly available API.

## Triggering notifications from a survey or experiment

Sending a notification to a participant or a group of participants can be triggered by the HTTP POST request embedded in an online survey or experiment. Most online study builders support JavaScript, so we provide a JavaScript code template on the Samply website (https://samply.uni-konstanz.de/api). For example, a researcher using the *lab.js study builder* (Henninger et al., 2022) or WEXTOR’s JavaScript plugin feature (Reips & Neuhaus, 2002; Reips & Shevchenko, 2023) can write a script to validate the user input and send the POST request if the input matches a predefined condition.

Research on determinants of relationship satisfaction provides a practical example of methodologies that can benefit from the Samply Stream API. Previous studies have employed experience sampling methods to collect data on relationship quality and shared experiences between partners. In these studies, prompts for self-report completion were delivered using one of two approaches: independent prompting, where notifications were sent separately to each partner (Debrot et al., 2018; Graham, 2008), or a yoked design, in which both partners received prompts at the same time (Li & Hui, 2019).

The Samply Stream API introduces an additional dimension to these studies through its event-contingent design capability. This feature allows researchers to implement a dynamic data collection process. For instance, in a study of couples, if one partner reports an event of interest (e.g., a quarrel), the API can automatically trigger a notification to the other partner with a survey about that specific event. This real-time, event-driven approach enables more targeted and contextually relevant data collection, potentially yielding richer insights into relationship dynamics than with other methods.

In the Samply interface, this type of study design requires assigning participants to groups (or dyads in the case of the study of couples), which can be done in two ways. First, participants can specify the group name in the Samply Research mobile app when they join the study. Alternatively, researchers can assign participants to a group on the *Samply* website (https://samply.uni-konstanz.de/groups). Once participants are connected through a group, notifications can be sent using Samply Stream API. If at least one of the group members reports an event, all the others can be notified and asked at the same time. In this way, the method improves on traditional individual self-reporting of events by reducing the likelihood of the event going unnoticed or being forgotten to report.

Self-initiated group notifications can help study events that occur infrequently or are likely to be missed (Moskowitz & Sadikaj, 2012). In the organizational setting, for example, these may be instances of altruistic behavior or injustice. The study of social interactions in couples may benefit from the ability to examine events such as marital conflict and its resolution, joy, lying, satisfaction, or guilt. In clinical studies of psychopathology (e.g., anxiety, borderline personality, bipolar disorder), events that can be recorded and linked to Samply Stream API may include instances of social anxiety or self-injurious thoughts. An application in therapy could focus on events such as sudden insights, binge eating, smoking relapse, or acute pain. An additional benefit for clinical and therapeutic applications may be the inclusion of family members or therapists in the group who receive notifications when the event of interest occurs.

## Triggering notifications with a public API

A public API provides access to a proprietary or open-source software application or web service. Typically, the result of an interaction with a public API is a piece of information, e.g., a weather forecast from the weather API. Some of the APIs can provide a stream of information, such as a chat application that transmits messages. If the API allows a user to interact with a stream of incoming information, a researcher can embed the POST request in the program that processes the incoming data. Information streams are most common on social networks, where users are constantly publishing new posts online.

Here, we describe web services that we worked with, although this list is just a few examples of a general approach. These are Twitter/X, Telegram, and RSS feed. The Twitter/X Stream API is an example of the API that provides researchers with information about published tweets in real time (Twitter Developer, 2024). The Stream API was on and off available for free for research purposes until recently (e.g., Reips & Garaizar, 2011), but since Twitter became a private company, this API is no longer available on the same terms as before.

Another example of a public API that can be integrated with Samply is the Telegram Bot API (Telegram, 2024). The Telegram Bot API provides a way to process information from public Telegram channels. A custom server application can listen to new posts from specific Telegram channels and trigger a notification when some predefined conditions are met. Although this API is potentially useful for studying activity in Telegram channels, its application remains limited, as official news agencies are not well represented in Telegram.

Finally, Samply Stream API can be integrated with an RSS feed. An RSS feed is a web feed that allows users and applications to receive updates from websites or blogs. A website, such as a news agency, provides RSS feeds as a way to publish regularly updated information. In the following, we describe how we used an RSS feed to test the Samply Stream API and investigate the perception of misinformation in real time. Because the news stories were modified using AI algorithms, we outline these first.

## AI algorithms

Large language models (LLMs), particularly ChatGPT (OpenAI, 2023), have made AI accessible through prompts written in plain conversational language. Previous research has demonstrated that LLMs are capable of performing various tasks, including generating narratives (de Lima et al., 2025) and fake news (Sun et al., 2024). Without delving into the technical details of the model’s mechanisms, we focus on its application in analyzing and modifying event-related information. This process generally involves constructing a prompt that includes the incoming information and sending it to the model via a ChatGPT API (OpenAI, 2023). The ChatGPT response can then be processed and presented to a participant. The advantage of this approach is its potential for automation, eliminating the need for manual editing or message sending. A simple example would be querying current weather conditions at a participant’s location using weather services and using this information to greet the user in a notification (e.g., “Good morning! Beware of heavy snow today”). A more complex example, which will be explored further in the feasibility study, involves real-time news modification by a ChatGPT model.

## Feasibility study

To demonstrate how the streaming method can be used in behavioral research, we have conducted a conceptual replication of the news perception study of Bago and colleagues (2020). They found that time pressure increased belief in false information by decreasing the deliberation, so one of our study goals was to replicate this finding. However, in contrast to the original study, in our experiment, the news headlines were streamed in real time rather than being prepared in advance. Also, participants evaluated the news on their smartphones throughout the day rather than participating in a one-time laboratory or online experiment, and the news messages were modified using AI as part of an experimental manipulation (original news, paraphrased news, and news with misinformation condition). We controlled how much deliberation people could engage in when evaluating the news headline by manipulating time constraints, by implementing experimental conditions with and without time limit).

We applied the same dependent measures as in previous research on misinformation, in which participants had to provide an accuracy rating and a sharing intention judgment (Bronstein et al., 2019). In addition, we assessed participants’ context and state, i.e., their location, presence of others, self-reported level of noise and distraction, and emotional state, as potential factors influencing the dependent variables. With regard to individual differences, as previous research suggested that delusion-like ideation, dogmatism, and religious beliefs were associated with belief in false information (Bronstein et al., 2019), we also included measures of these traits in our study.

To deliver news in real time to our study participants, we connected the Samply Stream API to the RSS feed of a news agency through a server-based application in the way that news stories were streamed to the study participants via smartphone notifications and presented to them for evaluation in an online survey. As this implementation is specific to an RSS feed, in the following, we describe the application in more detail.

## RSS feed integration with the Samply Stream API

We programmed a server-based NodeJS application^1^ to scan and process updates from a specific RSS feed, triggering notifications using a POST request to the Samply Stream API. The core functions of the application include periodically scanning the RSS feed using the npm package “rss-feed-emitter” (Deschamps, 2023) at a 1-min refresh rate, processing and modifying news messages with AI under one of the experimental conditions, storing the messages in a MongoDB database, and sending a POST request through the *fetch* command.

For this study, we selected the RSS feed of the German news agency Tagesschau (tagesschau.de—Die Nachrichten der ARD) for German-speaking participants and BBC News for English-speaking participants. Given that these feeds publish numerous news items daily and news can be released at any time, using the configuration parameters in the server-based application, we defined three time windows during which only the first news message was processed: 9:00–13:00, 13:00–17:00, and 17:00–21:00. This approach ensured that only three news items per day were sent to participants.

A typical RSS news message contains a header and the news content (maximum of three sentences). The text of the news message was pre-processed with the npm package “text-cleaner” to remove HTML (Gamble-Milner, 2024). After that, we used the Chat-GPT API (OpenAI model “gpt-4–0125-preview”) to modify the message to either contain misinformation or a paraphrased version of the original text (see Appendix B for the prompt). Once the original and modified versions were saved in the database, a POST request was sent to the Samply Stream API. To avoid transferring the text directly through the request and thus allow for a preview, we sent a web link generated for each text. When participants opened a link from the notification in their mobile browser, the text of the news was downloaded from that web link.

## Method

### Participants

The participants were Psychology students of the University of Konstanz recruited from the participant management platform Sona Systems. The participation was compensated with three hours of course credit. Additionally, a raffle was organized for the participants who completed over 80% of surveys, with the chance to win one of ten 50€ vouchers. We collected the dataset from 110 participants, 96 females, 11 males, and three undisclosed, *M*_age_ (*SD*) = 22 (3.71), who participated in at least one daily survey. Based on their preferred language, 93 participants completed the German version, and 17 participants completed the English version of the study.

### Research design

The study ran for 2 weeks for each participant, and started with the completion of the baseline survey, which collected information on socio-demographic variables and individual differences. The sampling schedule of daily surveys followed the event-contingent design, where the notifications were sent three times a day immediately after the news was published in the RSS feed. After 2 weeks, participants completed a debriefing survey, where they were asked for feedback and instructed on how to remove the mobile application from their device.

The daily surveys employed a within-subject experimental design to manipulate two factors: time constraint and news message type. The time constraint had two levels (with and without time limit), while the news message type had three levels (original, paraphrased, and with misinformation) (see Table 2). This resulted in a 2 × 3 within-subject factorial design, with each participant exposed to all conditions in random order. In the time constraint condition, participants had only 7 s to read the news before providing their judgments, following the method of Bago and colleagues (2020). The control condition imposed no time limit for reading the news.

**Table 2:** An example of the news message in three experimental conditions

Condition	News message
Original	Top Ukrainian general eyes leadership shake-up. Gen Oleksandr Syrskyi insisted the situation on Ukraine’s eastern front “remains difficult, but controlled”.
Paraphrased	Top Ukrainian general hints at minor adjustments in command. Gen Oleksandr Syrskyi stated the situation on Ukraine’s eastern front “continues to be challenging, yet manageable”.
With misinformation	Top Ukrainian general suspects foreign interference in leadership. Gen Oleksandr Syrskyi claims the situation on Ukraine’s eastern front “is deteriorating, amid uncontrolled chaos”.

### Measures

**Fig. 1:**
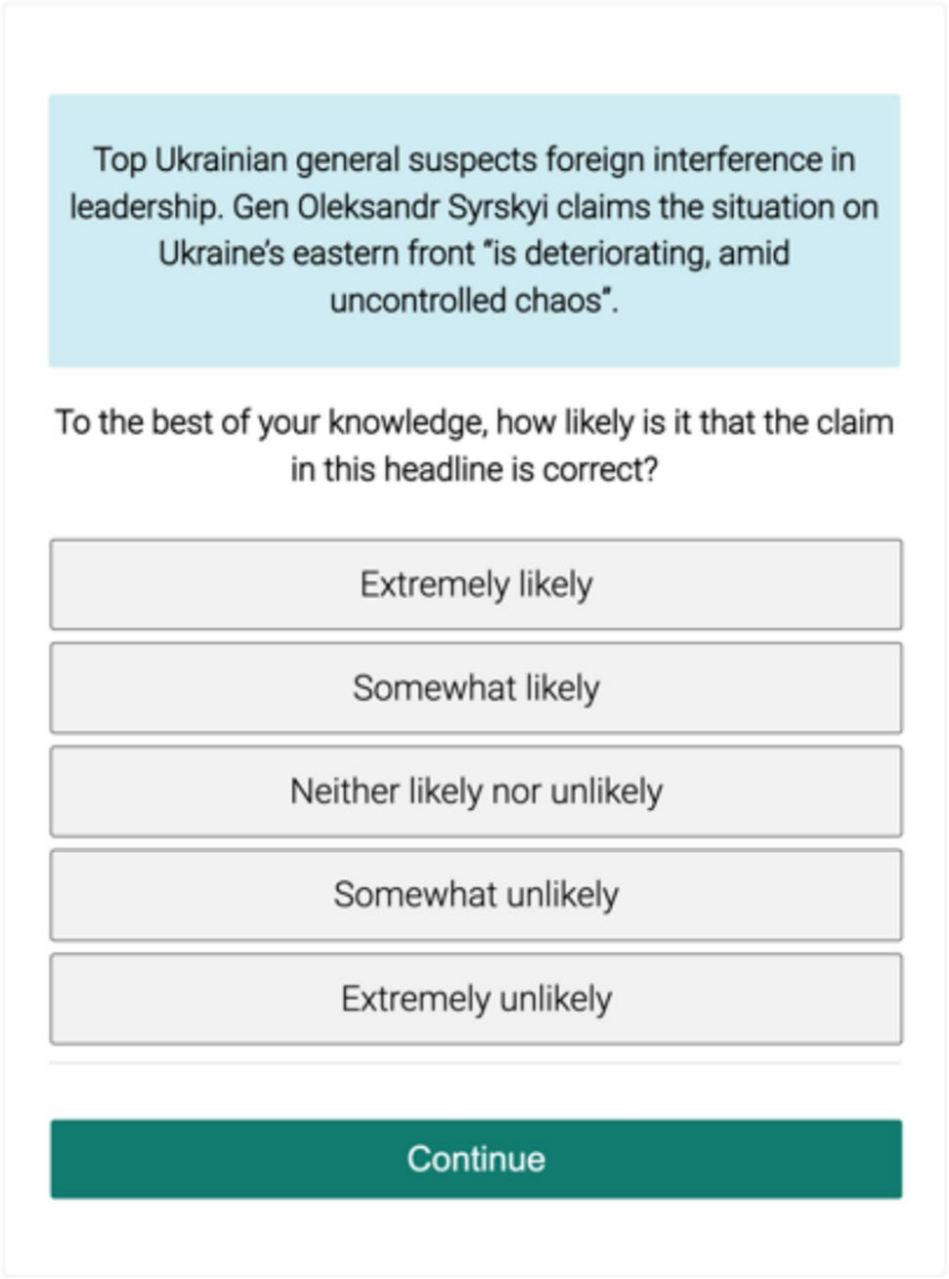
Screenshot of an experimental trial

All surveys were programmed in the lab.js experiment builder (https://lab.js.org, Henninger et al., 2022) and hosted on the Open Lab platform for data collection (Shevchenko, 2022). The baseline survey collected information on socio-demographic variables and individual differences (e.g., dogmatism, digital literacy, cognitive reflection). In the daily surveys, the participants answered questions about their current location, the presence of other people, the level of noise and distraction. After that, the participants were presented with the news and asked to evaluate its correctness using a Likert scale (from “Extremely likely” to “Extremely unlikely”, see Fig. 1), indicate their sharing intention (“no”, “maybe”, “yes”), and express their interest in reading the complete story (“no”, “yes”). We also asked participants whether the news message was readable (i.e., “The text is not possible to read”) and whether they had seen or heard about this news before (response options were “no”, “not sure”, and “yes”). At the end of each daily survey, the true nature of the news—whether original, paraphrased, or misinformation—was revealed, with an option to access the original story. In the final survey at the end of the study, participants were asked if they missed any notifications and if there were any technical or other problems during the study. Open-ended questions allowed participants to give us their feedback on the study. Finally, participants were asked to enter their e-mail addresses if they wished to participate in the raffle.

### Procedure

Participants who clicked on the study link were redirected to the webpage with the baseline survey. Here, participants (1) were informed about the study’s objectives, (2) were provided with an informed consent form, (3) completed the survey, and (4) were instructed on how to install and use the mobile application Samply Research. To anonymously connect participants’ data from different surveys, participants were shown a unique anonymous code, which they entered in the mobile application. This participant code was later recorded in all surveys that participants did through the mobile app. After joining the study in the mobile app, participants immediately received a test notification. The study lasted for 2 weeks for each participant, during which they received three daily notifications with links to surveys about real-time news. On Day 15, marking the end of the study, participants received a link to a debriefing survey through the app.

### Data protection

Self-report measures were obtained as part of the study, which posed no risk to participants and had already been used in previous studies. Participants were informed that they were free to interrupt the study at any time. Informed consent given in the mobile application *Samply Research* applied to data collected when using the application. Informed consent in the online surveys applied to data collected in the surveys. We used anonymous participant codes to connect the data from different surveys and the mobile application. The personal e-mail addresses used to log in to the mobile application were stored separately from the survey data, and the link between the e-mails and the survey data was destroyed after the study was completed. The personal e-mails provided for the raffle were stored separately from the survey data and deleted after the raffle had been conducted and the winners had been rewarded with vouchers.

### Data analysis

In the following section, we evaluate the feasibility of three interconnected aspects of our research: our approach to on-the-fly AI-based experimental manipulation of streamed information, the practical implementation of this approach, and the utilization of the Samply Stream API for these purposes. The data pertaining content hypotheses are reported elsewhere, as they fall outside the scope of this journal and article. Our focus was on examining how participants interacted with the mobile application and surveys, as well as the impact of the smartphone operating system (iOS, Android) on the software’s functionality.

All analyses were conducted using R version 4.3.2 (R Core Team, 2021). First, we conducted a dropout analysis using the dropR package (Reips et al., 2024) to evaluate participants’ overall compliance with the study instructions and to compare participant dropout for different smartphone operating systems. Second, we computed non-response rates and response latencies, then employed mixed-effects models using the lme4 package (Bates et al., 2014) to determine the association between compliance and various factors, including individual characteristics, smartphone-specific variables, and situational factors. To evaluate the feasibility of the AI-based manipulation of news, we compared the amount of non-readable and familiar news stories in the original, paraphrased and misinformation conditions. Finally, we reviewed the feedback provided by participants at the end of the study to identify any systematic technical issues related to the delivery of notifications. The data and analysis script are available at OSF (https://osf.io/2tw9u/).

## Results

We analyzed data from 108 participants, focusing on 66 iOS and 42 Android smartphone users, not including users on laptop and desktop computers (one Linux and one Mac OS X user). There were no statistically significant differences in age or gender between iOS and Android users (*p*s > 0.05). On average, during 2 weeks, participants completed 35 daily surveys (*SD* = 7.24), which corresponds to 83% of all daily surveys. A substantial proportion of participants (94%) completed more than 50% of daily surveys (100% of Android users and 91% of iOS users). Android users provided more responses than iOS users, with Android users completing an average of 38 surveys (*SD* = 4.49), compared to iOS users’ 33 surveys (*SD* = 7.95), *t*(104.76) = 4.31, *p* < 0.001.

## Dropout

**Fig. 2:**
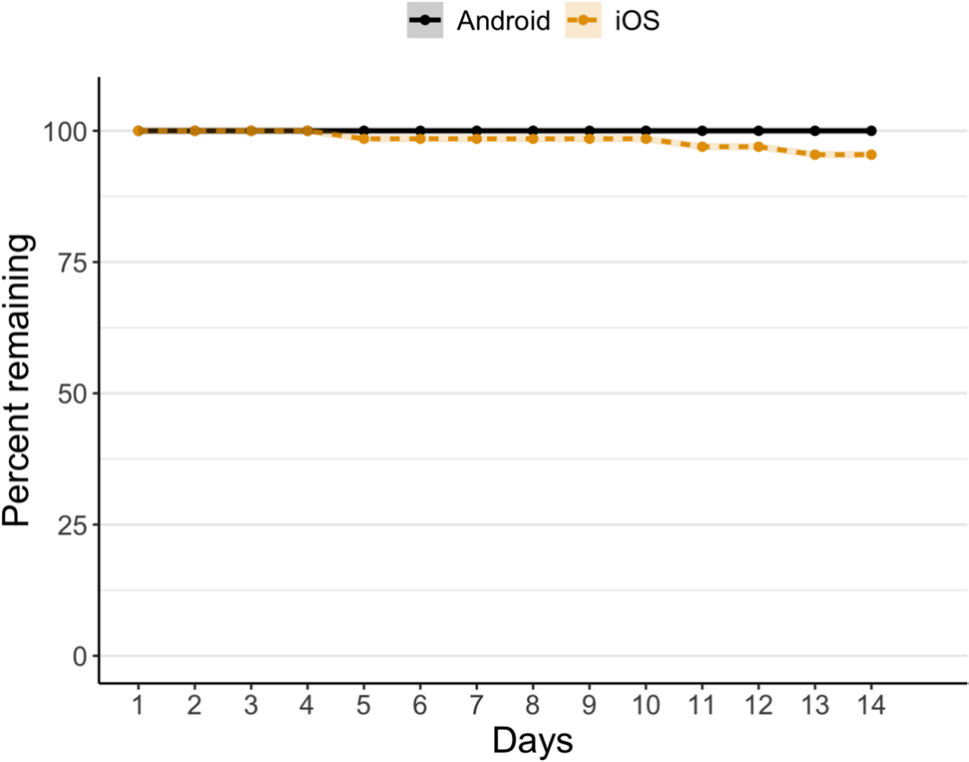
The percent of remaining iOS and Android users at each day of the study

By the end of the 2-week study, three iOS (5%) and no Android users had dropped out from the study (see Fig. 2 for the dropout curves during the study). A chi-square test was conducted to compare the dropout rates between iOS and Android users. The results showed no statistically significant difference in the number of dropouts at the study’s end, *X* ^2^(1, *N* = 108) = 1.96, *p* = 0.28.

### Non-response analysis

Using response as a dependent binary variable (0 for non-response, 1 for response), we estimated a logistic mixed model that included several individual and situational predictors. Each participant was modeled with a random intercept. The individual variables included gender and smartphone operating system (iOS vs. Android). The situational variables, which varied for each measurement, included whether it was a weekend or not, time of day (morning, afternoon, evening), and the day of the study. With each additional day in the study, the probability of responding to a notification decreased, OR = 0.96, *SE* = 0.01, *p* < 0.001 (see Table 3). Additionally, on average, iOS users had a significantly lower response rate than Android users, with iOS users responding 77% of the time compared to Android users’ 89%, OR = 0.33, *SE* = 0.08, *p* < 0.001.

**Table 3:** Estimates for the variables influencing the probability of response

	Response			
Predictors	Odds ratios	*SE*	CI	*p*
(Intercept)	18.85	4.27	12.09–29.38	< 0.001
Gender [male vs. female]	0.93	0.36	0.43–1.99	0.85
OS [iOS vs. Android]	0.33	0.08	0.20–0.53	** < 0.001**
Day	0.96	0.01	0.94–0.98	** < 0.001**
Time of day [afternoon vs. morning]	0.98	0.09	0.82–1.17	0.81
Time of day [evening vs. morning]	0.96	0.36	0.46–1.99	0.91
Time of week [weekend vs. weekday]	0.89	0.08	0.74–1.07	0.23
Random effects				
σ^2^	3.29			
τ_00_ _participant_	1.11			
ICC	0.25			
N _participant_	105			
Observations	4447			
Marginal *R*^2^ / Conditional *R*^2^	0.07/0.30			

**Fig. 3:**
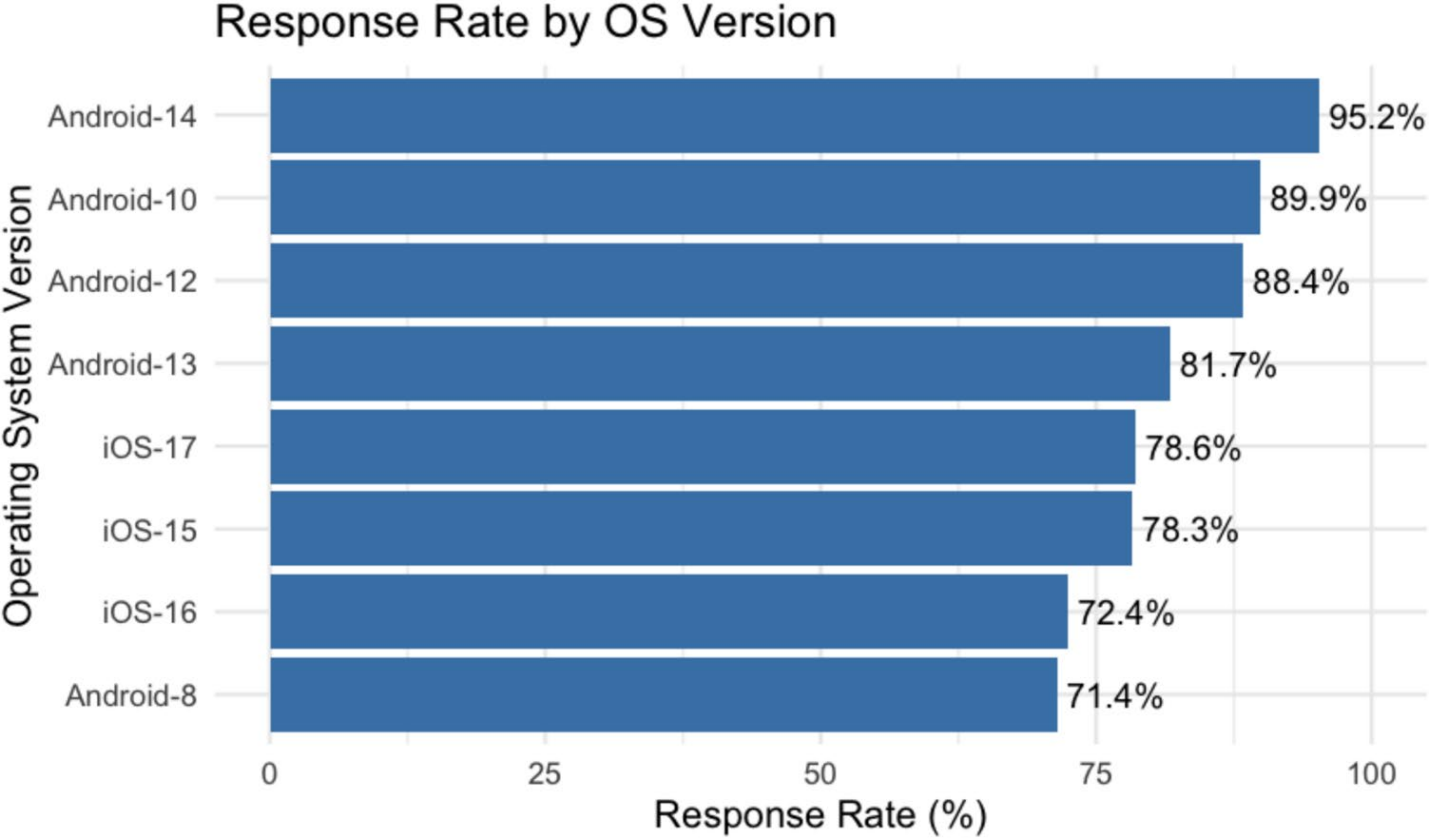
Response rate for each operating system version

The analysis of operating system versions revealed interesting patterns in response rates that differed between iOS and Android. As shown in Fig. 3, Android devices generally exhibited higher response rates compared to iOS, with a trend of increasing response rates correlating with more recent Android versions.^2^ Specifically, the response rate for Android devices ranged from 71.4% for Android 8 to 95.2% for the latest Android 14. In contrast, iOS devices showed consistently lower response rates across different versions, with minimal variation between versions. For instance, iOS 15 and iOS 17 had similar response rates of 78.3% and 78.6%, respectively.

### Response latency analysis

For the surveys for which responses were collected, we calculated the response latency as the time difference between when the notification was sent and when the participant opened the survey. While response latency does not directly indicate software functionality, lower latency can be interpreted as an indicator of higher compliance (Boemo et al., 2022), a factor generally valued by researchers aiming to minimize the temporal gap between notification and self-reporting. We used a linear mixed model with predictors similar to those from the non-response analysis, along with additional predictors extracted from participants’ responses, such as noise, distraction, and current location (see Table 4). With each additional day in the study, the latency increased, *b* = 0.43, *SE* = 0.16, *p* = 0.008. Afternoon response latencies were significantly shorter than those in the morning (*b* = – 5.20, *SE* = 1.39, *p* < 0.001). Participants responded slower on a weekday compared to weekends (*b* = 3.38, *SE* = 1.48, *p* = 0.022).

**Table 4:** Estimates for the variables influencing the response latency

	Response latency			
Predictors	Estimates	*SE*	CI	*p*
(Intercept)	32.37	3.96	24.60–40.14	** < 0.001**
Gender [male vs. female]	0.02	4.97	– 9.73 to 9.77	0.99
OS [iOS vs. Android]	0.38	3.02	– 5.54 to 6.30	0.90
Day	0.43	0.16	0.11–0.75	**0.008**
Time of day [afternoon vs. morning]	– 5.20	1.39	– 7.92 to – 2.48	** < 0.001**
Time of day [evening vs. morning]	– 11.18	6.38	– 23.69 to 1.32	0.08
Time of week [weekend vs. weekday]	3.38	1.48	0.48–6.27	**0.022**
Noise	0.76	0.81	– 0.82 to 2.35	0.35
Distraction	– 1.98	1.27	– 4.47 to 0.51	0.12
Location [home vs. not home]	– 0.75	1.68	– 4.05 to 2.54	0.66
Random effects				
σ^2^	1556.49			
τ_00_ _participant_	176.27			
ICC	0.10			
N _participant_	105			
Observations	3642			
Marginal *R*^2^ / Conditional R^2^	0.008 / 0.109			

The response latency is provided in minutes.

### Comparison of news messages evaluations

We examined the participants’ responses across three experimental conditions: Original news, paraphrased news, and news with misinformation (see Table 5). Our analysis focused on technical readability, news familiarity, and participants’ evaluations of the content.

**Table 5:** Evaluation of news in different experimental conditions

	Original news	Paraphrased news	News with misinformation
*N*	1260	1295	1257
Number of non-readable news items (%)	12 (1.0%)	18 (1.4%)	16 (1.3%)
Number of times that participants did not hear or see the news story before (%)	918 (73%)	947 (73%)	1052 (84%)
Perceived correctness, Mean (SD)	3.62 (1.08)	3.60 (1.08)	2.63 (1.20)
Sharing intention, Mean (SD)	0.12 (0.32)	0.11 (0.31)	0.07 (0.26)
Intention to read, Mean (SD)	0.29 (0.32)	0.29 (0.31)	0.32 (0.26)

### Technical processing and familiarity

A minimal proportion of news items (1.2%, 46 out of 3812) were reported as unreadable, with no significant difference between conditions, *F*(2, 327) = 0.21, *p* = 0.81. While 73% of participants reported no prior exposure to the news items overall, the unfamiliarity rate was significantly higher for misinformation-injected news (84%) compared to original and paraphrased news (73%), *t* = – 3.72, *p* < 0.001, and *t* = – 3.84, *p* < 0.001.

### Content evaluation and behavioral intentions

Statistical analysis revealed significant differences in perceived news correctness across conditions, *F*(2, 3809) = 335.5, *p* < 0.001, with post hoc Tukey tests showing that participants rated misinformation-containing news as less correct than both original and paraphrased versions (*p*s < 0.001). Sharing intentions also varied significantly between conditions, *F*(2, 3809) = 10.73, *p* < 0.001, with lower willingness to share misinformation compared to original and paraphrased news (*p*s < 0.001). Reading intentions, however, remained consistent across all conditions, *F*(2, 3809) = 0.39, *p* = 0.68.

### Analysis of user feedback

We collected 55 responses to the open question in the feedback survey, which asked participants to indicate whether they experienced any technical problems during the study. Out of these, 45 responses explicitly stated that there were no technical problems. Seven participants in the time limit condition mentioned that the text of the news was displayed too quickly, leaving insufficient time to read it. However, this was intentional for the time limit condition and is not related to the functionality of the Samply Stream API. Three participants reported that they were missing a confirmation of completed surveys within the app, expressing uncertainty about whether their entries were saved (“I was not sure if it was saved what I entered”). Another three participants felt that they sometimes did not receive notifications (“Sometimes I didn’t get a notification to participate in the survey and when I realized it, the link had expired”), which might be related to the type of operating system they were using (in all three cases, it was iOS 16). However, all other participants using iOS 16 either did not report any problems or explicitly stated that there were no technical issues during the study. Two participants mentioned having problems receiving notifications, possibly due to traveling or being in a different time zone. One participant tried to return and continue the survey after clicking on the news link on the last page, which is supposed to end the survey (“I'm not sure if I just didn't understand the navigation”). Another participant noted that clicking on the notification opened a new tab in the mobile browser, which eventually cluttered their screen (“At some point, I had 50 + tabs open”).

## Discussion

The main goal of this article is to introduce a new streaming method and AI-based on-the-fly modification that can enrich the field of behavioral and social research. This article presents the method and software and conducts a feasibility study to assess its effectiveness. The feasibility study demonstrated that the dropout and non-response rates are comparable to those in other longitudinal studies (Eisele et al., 2020; Rintala et al., 2020).

Regarding differences between smartphone operating systems, the dropout rate was similar between iOS and Android. However, the response rate was lower for iOS users compared to Android users. While we cannot rule out systematic differences between users of iOS and Android devices (e.g., Götz et al., 2017), the lower response rate for iOS users might be related to privacy defaults and technical limitations, particularly issues with the delivery of notifications. Potential reasons include the Focus Mode (e.g., Do Not Disturb), which blocks notifications and Low Power Mode, which can reduce background activity, including notifications.

Despite the lower response rate for iOS users, the response latency was similar between iOS and Android users, indicating no systematic differences in compliance. This supports the idea that non-delivered notifications on iOS could be the primary reason for the lower response rate. Future studies can address this issue by explicitly instructing participants not to disable notifications and by monitoring whether the app is still allowed to send notifications. Given a lower response rate on smartphones with an older operating system version, it is recommended to remind participants to update their operating system, which also offers better protection against malware attacks.

We observed that the response rate decreased with each day of the study while response latency increased, corresponding to dropout rates and sample attrition. Response latencies exhibited a distinct diurnal pattern, with significantly longer delays during morning hours compared to afternoon periods. This morning-specific pattern aligns with existing research. Rintala and colleagues (2023) reported higher disturbance by study prompts in the morning, while Stieger and Reips (2019) found that people generally report lower well-being during morning hours.

Analysis revealed shorter response latencies during weekdays compared to weekends, likely reflecting participants’ higher smartphone engagement during work or study hours versus leisure time. Contrary to expectations, noise and distraction were not related to response latency, suggesting that participants in more distracted and noisy environments did not postpone their answers.

With regard to the use of AI algorithm for modifying the incoming information in real time, the results of our study provide evidence for the effectiveness of on-the-fly changes. The consistency in non-readability rates across all conditions (original, paraphrased, and misinformation-injected) demonstrates that the AI-generated modifications did not introduce significant technical errors or compromise the overall readability of the texts. This indicates that our AI method successfully maintained the linguistic quality and coherence of the news articles while manipulating their content. Moreover, the significant difference in perceived familiarity between the misinformation-injected condition and the original/paraphrased conditions (84% vs. 73% unfamiliarity) suggests that the AI effectively introduced novel elements that were not readily recognizable to participants. At the same time, participants demonstrated preserved critical evaluation abilities, as reflected in their lower perceived correctness ratings and reduced sharing intentions for misinformation-injected news. This pattern of results indicates that while the AI-generated modifications were seamlessly integrated from a technical perspective, the semantic changes remained detectable to attentive readers. These findings collectively support the viability and effectiveness of using AI to manipulate incoming information “on the fly”, opening up new avenues for research in areas such as media literacy, misinformation studies, and AI-assisted content generation.

In summary, the streaming method allows for the real-time transmission of information to participants and can be applied across various domains (see Table 6 in Appendix C). We demonstrate the versatility and power of using real-time data streaming combined with mobile surveys or experiments to gather immediate, contextually relevant data across diverse fields. Additionally, our feasibility study successfully implemented AI-based modifications of news headlines in real-time. This demonstrates the potential for dynamic, AI-driven content manipulation in behavioral research.

## Conclusion

We have successfully implemented and tested a new streaming and AI-based on-the-fly modification method, offering new possibilities for behavioral and social research. This approach enables researchers to collect contextually relevant data in real-time that provides a powerful tool for studying human behavior in naturalistic settings and thus likely enhances the external validity of the research. The method shows promise for various applications, including public opinion research, healthcare, marketing, and environmental monitoring. It addresses the limitations of traditional laboratory experiments by allowing for the study of reactions to current events and personalized content. Importantly, the tools and code for implementing this method are freely available to researchers. The Samply application, including the Samply Stream API, can be accessed at no cost. The server-based application for RSS feed integration and AI manipulation is open-source and available on GitHub. Its accessibility encourages further development and application of the method across diverse research domains. In conclusion, the streaming method with AI-based manipulation represents a significant advancement in experience sampling methodology and in combining ESM with experiments, offering researchers new ways to capture and analyze human behavior and decision-making in real-world contexts.

## Data Availability

The data and analysis script are available on OSF (https://osf.io/2tw9u/).

## Appendix A

**Tutorial on Samply Stream API** 3

**Step 1. Select recipients**

Notifications can be sent to a specific participant, to a specific group of participants, or to all participants of the study. To send an individual notification via the Samply Stream API, researchers should specify the participant ID in the POST request. To send a group notification, researchers use the group ID. There are two ways to assign the participants into groups: assignment by participants and assignment by the researcher.

Assignment by participants: participants can be asked to provide the group’s name when joining the study. The instructions about the group name can be created and edited by the researcher (e.g., “Please enter the name of the group here” or “Enter your group code”). To do that, on the following page, https://samply.uni-konstanz.de/projects, click on the pencil icon on the study. You will then be redirected to the edit page, where you can click the checkbox option “Ask participants to enter a group code in the mobile application when joining your study” to edit the instructions. After joining the study, the group’s name will be displayed in the mobile app.

Assignment by the researcher: participants can be assigned into groups on the researcher’s website. The prerequisite here is that the participants are already registered for the study. Groups can be created at https://samply.uni-konstanz.de/groups by entering the group name and selecting participants by clicking on them. The groups can contain as many participants as you wish. However, each participant can only be in one group. Changes in the group or in the group name will be displayed for the participant inside the mobile app.

**Step 2. Generate the authorization token**

The next step is to create an authorization token with a specific expiration date. This can be done on the page “Groups” under “Notify API” (https://samply.uni-konstanz.de/api) by selecting the expiration date and clicking on the “Reset token” button. This token authorizes participants to send notifications to other participants when certain events occur. The expiration date is used for additional security, so you control how long the notification token is valid.

**Step 3. Embed the POST request in your software**

At the very bottom of the same page (https://samply.uni-konstanz.de/api), you will see a code snippet that can be used to trigger the notifications. This code can be implemented as an HTTP POST request in any online software that supports JavaScript.

The URL where the POST request is sent should remain the same: https://samply.uni-konstanz.de/api/notify. The data object consists of information that helps identify to whom the notification should be sent.

- The *project ID* is unique for each study and will be shown in the code provided on the website.
- *Group ID* and *Participant ID*. Participant and group IDs are displayed on the website either on the group page (https://samply.uni-konstanz.de/groups) or on the participant page (https://samply.uni-konstanz.de/users).

**Table:**

Participant ID	Group ID	Notification recipients
Specified	Specified	All members of the group except the participant will receive the notification
Unspecified	Specified	All members of the group will receive the notification
Specified	Unspecified	Only the specific participant will receive the notification
Unspecified	Unspecified	All participants in the study will receive the notification

- • The *token* value should be equal to the value of the authorization token.
- • The *title*, *message*, and *url* parameters define the content of the notification that will be displayed to the participants.
- • It is recommended to include query parameters (SAMPLY_ID, PARTICIPANT_CODE, GROUP_CODE, etc.) in the URL. These parameters will be replaced with actual participant-specific values when a notification is sent. That will allow us to record this information on the survey side by extracting the values from the URL.
- • The *expireIn* parameter defines in how many minutes the notification link should expire.

**JavaScript code to use the Samply Stream API to send a notification**

**Methodological issues and recommendations**

**Excluding some participants from being notified**

Sometimes researchers would like to exclude some participants from being notified about an event. If a response in the survey triggers the notification, the participant who gave the response should not be notified about that. For example, if one partner reports a conflict and fills out a survey, only the other partner should get notification with the invitation to fill in the survey. Samply Stream API allows to specify the participant that should not be notified in case of an event. It is done by providing both the participant’s Samply ID in the POST request in the field *participantID* and the group ID in the field *groupID*.

**Security**

As participants install the app and allow notifications from it, they permit researchers to send them notifications at any time. To protect the Samply Stream API from a potential security breach and abuse, such as sending spam messages to study participants, we implemented private tokens with an expiration date. To use the Samply Stream API, the private researcher token should be included in the POST request. This token can be generated on the Samply website. We also recommend researchers set up an expiration date for the token, so notifications will not be sent after the token has expired. The previous token can always be invalidated by generating a new one. In case of a security breach and someone getting to know the private token, a researcher can invalidate the old compromised token.

**Passing additional information from external APIs to Samply notification**

Researchers can customize the title, body, and URL of the Samply notification. Sometimes researchers need to include the information from an external API. For example, in the current study, we passed the ID of the news story via the Samply notification to the online survey. This can be done via query parameters that are included in the link (e.g., https://survey.example.com?newsid=12345 …). The researcher should avoid putting private and sensitive information there, as the link can be saved in browser history or be available in the server logs of the survey website.

**Further customization**

The Samply Stream API is intentionally designed with a minimalist and universal approach. If researchers need to further customize a POST request (e.g., trigger a notification if special conditions are met), an optimal solution is to implement conditional logic in the client application that interfaces with the Samply Stream API. This approach offers several key advantages: it maintains proper separation of tasks by allowing the client application to handle specific rules while the API maintains its universal character, ensures scalability as each research project can implement its unique conditions without modifying the core API, and improves maintainability since updates to conditional logic can be made without server-side changes.

## Appendix B

### Prompt for Chat-GPT

“We are conducting a study on the perception of fake news. For this particular situation, you need to come up with two short (maximum of three sentences) modified messages based on the original message, which will be provided. The language of the modified messages should match the language of the original message. So, if the original message is in English, the modified messages should be in English. If the original message is in German, the modified messages should be in German. The modified messages should have the same length as the original message. The first modified message should be as close as possible to the original news, so it should not contain misinformation. The second modified message should be fake news, so it should contain misinformation. You must respond with only the text for these two messages. Do not include any headers for modified messages. The original message is {placeholder for the original news message}.”

## Appendix C

**Table 6:** Application areas of the streaming method

Field	Topic	Example of research question
Social Media and Communication	Trending topics analysis	Send updates on trending social media topics and measure how opinions shift over time or in reaction to new information
	Effect of social media on opinions	Explore how exposure to various types of social media content influences beliefs and attitudes
Media and Journalism	Viewer engagement	During live events, news broadcasts, or streaming of shows, collect viewer perceptions and engagement levels to understand what draws and maintains interest
	Impact of news	Evaluate how different types of news stories (e.g., crime, economic issues) impact the public’s emotions and perceptions
Market Research and Consumer Behavior	Advertising effectiveness	Measure immediate reactions to advertising campaigns in real time
	Shopping behaviors	Understand how real-time promotions or information about products influence consumer purchasing decisions
Public Opinion and Political Science	Election monitoring	Stream live political debates or election results, and gather instant reactions and opinions from viewers
	Policy feedback	Disseminate new policy proposals to a broad audience and collect instant feedback, aiding in policy adjustment and public engagement
Financial Markets	Investor sentiment analysis	Provide real-time stock market updates and gather immediate feedback from investors about their perceptions and investment strategies
	Market trends	Track how news affects market sentiment and decisions in real time, helping in the study of market dynamics
Health and Wellness	Public health monitoring	Send updates about health alerts (like pandemic outbreaks) and assess public reactions and knowledge levels instantly
	Behavioral interventions	Test the effectiveness of real-time health interventions, such as reminders to take medications or prompts to engage in physical activity
Environmental and Weather-Related Research	Climate change perception	Stream information about climate events or new research findings and measure public concern and awareness
	Disaster response and preparedness	During natural disasters, provide real-time updates and surveys to assess the effectiveness of communication and the preparedness of communities
Educational Research	Learning and engagement	Deliver educational content and immediately quiz users to gauge understanding and retention
	Feedback on educational tools	Collect real-time feedback from students using new educational apps or technologies

## Footnotes

1 The software code is available at GitHub https://github.com/Yury-Shevchenko/samply-streamer.

2 Older smartphone models were probably running outdated software versions. However, we did not record the specific device types, so this assumption cannot be verified.

## Code availability

Samply is available free of charge as a website and mobile app. Researchers can set up studies using the website interface (https://samply.uni-konstanz.de/). The “Samply Research” app for participants can be downloaded from Apple and Google app stores. The experiment’s software code, using the Samply Stream API, is available on GitHub (https://github.com/Yury-Shevchenko/samply-streamer).

## Declarations

### Open practices statement

The data and materials for the experiment are available on OSF (https://osf.io/2tw9u/). The experiment was not preregistered as it aimed to evaluate software implementation feasibility rather than hypothesis testing.
